# Crescentic glomerulonephritis in children: short-term follow-up predicts long-term outcome

**DOI:** 10.3389/fped.2023.1206168

**Published:** 2023-08-25

**Authors:** Pei Zhang, Xiao Yang, Chun-lin Gao, Wei Wu, Zheng-kun Xia

**Affiliations:** ^1^ Department of Pediatrics, Jinling Hospital, Affiliated Hospital of Medical School, Nanjing University, Nanjing, China; ^2^ Department of Pediatrics, Longgang District Center Hospital of Shenzhen, Shenzhen, China

**Keywords:** crescentic glomerulonephritis, pathological lesions, prognosis, end-stage kidney disease, children

## Abstract

**Background:**

Crescentic glomerulonephritis (CrGN) is a relatively rare but severe condition in childhood with the clinical feature of rapidly progressive glomerulonephritis (RPGN). The aim of this study is to investigate the clinicopathological features and prognosis of CrGN in children.

**Methods:**

We retrospectively analyzed the clinical and laboratory data, renal pathological results, treatment, and outcome of 147 CrGN in two Chinese pediatric nephrology centers.

**Results:**

Among the 147 children, there were 22 cases of type I (15.0%), 69 cases of type II (46.9%), and 56 cases of type III (38.1%). The mean percentages of crescents in CrGN I, II, and III were 85.3%, 68.7%, and 73.6%, respectively. The children with type I CrGN presented with more severe clinical manifestations and pathological lesions. The 3-month cumulative renal survival rates of types I, II, and III CrGN were 66.3%, 93.6%, and 75.6%, respectively. The 1-year cumulative renal survival rates of types I, II, and III CrGN were 56.9%, 85.3%, and 73.1%, respectively, and the 5-year cumulative renal survival rates of types I, II, and III CrGN were 33.8%, 73.5%, and 47.1%, respectively. The Kappa Consistency Test between the 3-month and 1-year total renal survival (82.1% vs. 74.7%) of the children was 0.683 (*P* < 0.001), and between the 1-year and 5-year total renal-free survival (78.3% vs. 69.1%) of the children was 0.476 (*P* < 0.001). The Bowman's Capsule Rupture (BCR), crescent, interstitial inflammation, and interstitial fibrosis/tubular atrophy (IF/TA) score were predictors of end-stage kidney disease (ESKD) risk but BCR showed better predictive value for ESKD than interstitial inflammation score (*P* = 0.027) and IF/TA score (*P* = 0.047).

**Conclusion:**

Patients with type I tended to have the worst renal survival rates. The three-month renal prognosis could partially reflect the 1-year renal prognosis, and the 1-year mortality rate could partially reflect the 5-year mortality rate of children with CrGN.

## Introduction

Crescentic glomerulonephritis (CrGN) is a type of glomerulonephritis that is induced by a variety of causes and result in a sudden and progressive decline in renal function, which is referred to as rapid progressive glomerulonephritis (RPGN). CrGN is defined histopathologically by crescents in 50% or more of glomeruli. CrGN is classified into different subtypes: Type I: anti-glomerular basement-membrane (anti-GBM) disease, which is mediated by anti-glomerular basement membrane (GBM) antibodies, and the immune complex deposits linearly along the GBM; Type II: CrGN caused by deposition of the immune complex, such as immunoglobulin A nephropathy (IgAN), postinfectious glomerulonephritis (PIGN), lupus nephritis (LN), Henoch-Schonlein purpura nephritis (HSPN), membranoproliferative glomerulonephritis (MPGN); and Type III: pauci-immune glomerulonephritis (little or no staining for immunoglobulins or complement), which is mediated by anti-neutrophil cytoplasmic antibody (ANCA). A few patients show ANCA and anti-GBM antibody double-positive, and clinicians have defined them as type IV. Type V is considered ANCA-negative, pauci-immune glomerulonephritis (5% to 10% of cases) (1). However, the two types in clinic had not been widely accepted.

CrGN is a relatively rare disease; it accounts for 1.6% to 10% of glomerulopathy confirmed by renal biopsy (2). The primary signs of CrGN are hematuria, albuminuria, nephritic sediment, decreased estimated glomerular filtration rate (eGFR), and oliguria. The severity of the disease is related to the percentage of crescent formation. Patients with crescents in more than 80% of glomeruli may not respond well to therapy and tend to present with advanced renal failure. CrGN with a focal lesion with more than 50% normal glomeruli has a more favorable prognosis with almost 90% renal survival after 5 years of follow-up, whereas more than 50% of glomeruli with cellular crescent has a less favorable prognosis (1). However, reports on the prognosis for children with CrGN are scarce. Therefore, we retrospectively analyzed the clinical and pathological features and outcomes of children with CrGN diagnosed by renal biopsy from Jan 2008 to Jan 2018 at two large Chinese diagnostic and treatment centers for children's kidney disease.

## Materials and methods

### Study design and setting

Children with newly diagnosed CrGN in the Department of Pediatric, Jinling Hospital, Affiliated Hospital of Medical School, Nanjing University and Longgang District Center Hospital of Shenzhen, from Jan 2008 to Jan 2018 were recruited consecutively into the retrospective study. All the patients were <18 years old. The diagnosis was based on an examination of renal biopsy tissue containing at least 10 glomeruli. The ethics committees of Jinling Hospital and Longgang District Center Hospital of Shenzhen approved the study and informed parental consent was obtained. The study conformed to the principles outlined in the Declaration of Helsinki and was approved by the Ethical Committee of Jinling Hospital (number: 2018JLHGKJDWLS-184) and Longgang District Center Hospital of Shenzhen (number: ZSSOM 2018-0177).

### Clinical and biochemical measurements

At the time of renal biopsy, baseline data were collected from the hospital's electronic medical record (EMR) system, including age, sex, disease duration, clinicopathological, hematology, and urine examination. The treatment date and pathological features were also obtained from the EMR system. The eGFR was calculated using the modified Schwartz formula (3). Acute kidney injury (AKI), acute kidney disease (AKD), and chronic kidney disease (CKD) definitions were based on the Kidney Disease: Improving Global Outcomes (KDIGO) (4).

### Definition

According to the extent of interstitial inflammation, i.e., <1%, 1%–25%, 25%–50%, and >50%, the scores were semi-quantitatively graded as 0, 1, 2, and 3, respectively. For tubulitis, score 1: 0–9 inflammatory cells/tubules, score 2: 10–14 cells/tubules, and score 3: 15-or more cells/tubules in the most affected region (5). The extent of interstitial fibrosis/tubular atrophy (IF/TA) was graded into four categories: score 0: lesion < 5%; score 1: 5% ≤ lesion < 20%; score 2: 20% ≤ lesion < 50%; and score 3: lesion ≥ 50% (6). The extent of sclerosis was evaluated and scored as follows: score 0: <10%, 1: 10–25%, 2: 26–50%, 3: >50%, and the arteriosclerosis lesions were scored as follows: score 0: intimal thickening < thickness of media, score 1: intimal thickening thickness of media) (7).

The segmental crescent volume was less than 50% of the renal capsule, and the spherical crescent volume accounts for more than 50% of the renal capsule. Cellular crescents were defined as cells occupying 50% of the extracapillary lesion, and fibrocellular crescents were defined as extracapillary lesions consisting of cells and extracellular matrix, with cells < 50% and matrix < 90%. A fibrous crescent was defined as > 10% of the capsular perimeter being covered by a lesion composed of ≥ 90% of the matrix. Cellular/fibrocellular/fibrous crescents were calculated according to the relative ratio. Crescent scores = (percent of circumferential cellular/mixed/fibrous crescents) + 1/2(percent of segmental cellular/mixed/fibrous crescents) (8).

### Outcome measures

The renal endpoint was end-stage kidney disease (ESKD), and the ESKD-free survival endpoint was defined as death from any cause. ESKD was defined as kidney failure that reaches an eGFR ≤15 ml/min/1.72 m^2^ or needs maintaining renal replacement therapy (RRT) for more than 3 months.

### Statistical analysis

All analyses were performed using SPSS (version 24.0, SPSS Inc, Chicago, IL, USA). Continuous variables were described as mean and standard deviation or median [interquartile range (IQR)], and differences between groups were analyzed using a two-factor analysis of variance or a non-parametric test. Categorized variables were described as percentages and were analyzed using the χ^2^ test. The prognosis was evaluated using Kaplan-Meier curves, and the log-rank test was used to test the two curves' differences. The association of variables with ESKD was assessed with univariate and multivariate Cox proportional hazard regression models. The Kappa Consistency Test was used to describe the association between the 3-month, 1-year and 5-year outcomes. *P* < 0.05 was considered statistically significant.

## Results

### Baseline characteristics of children with CrGN

In the present retrospective study, CrGN accounted for 1.1% (147/1, 3,125) of the total number of nontransplant renal biopsies during the study period. Among the 147 patients, 22 (15.0%), 69 (46.9%), and 56 (38.1%) children were classified as type I, type II, and type III, respectively. The patients had a variety of underlying conditions or diseases. The etiology was immune complex in 19 cases, anti-GBM antibody disease in 22 (15.0%) cases, IgAN in 23 (15.7%) cases, LN in 19 (12.9%) cases, HSPN in 14 (9.5%) cases, PIGN in 8 (5.4%) cases, MPGN in 5 (3.4%) cases, ANCA-associated glomerulonephritis (AAGN) in 54 (36.7%) cases, and ANCA-negative pauci-immune CrGN in 2 (1.4%) cases.

The baseline characteristics of the children were presented in Table 1. The mean age was 13.45 ± 3.75 years, and 65 cases (44.2%) were male. The percentage of oliguria or anuria in type II was lower than in type I and type III, respectively (*P* < 0.05, *P* < 0.01). Among the total patients, 11.5% of patients could be classified as stage 1 AKI, 23.7% as stage 2 AKI, and 64.9% as stage 3 AKI. The percentage of stage 3 AKI in type I was higher than in type II and type III, respectively (*P* < 0.01). The levels of procalcitonin (PCT), interleukin-6 (IL-6), and proteinuria in type I were lower than in type II and type III, respectively (*P* < 0.05, *P* < 0.01). The level of serum creatinine (Scr) in type I was higher than in type II and type III, respectively (*P* < 0.05, *P* < 0.01), and eGFR was lower than in type II (*P* < 0.01). The level of urine red blood cell count (RBC) in type III was lower than in type I and type II, respectively (*P* < 0.01). The levels of urine N-acetyl-β-D-glucosidase (NAG) and retinol-binding protein (RBP) in type I were higher than in type II and type III, respectively (*P* < 0.01). Of the patients with type III CrGN, 96.4% were serum ANCA positive. Circulating anti-GBM antibodies were detected in all patients with type I CrGN, among whom 1.4% were positive for both ANCA and anti-GBM. There were 131 (89.1%) patients treated with immunosuppressive therapy, 91 children (61.9%) received continuous renal replacement therapy (CRRT) treatment, and the percentage of CRRT treatment in type II was lower than in type I and type III, respectively (*P* < 0.01) (Table 1).

**Table 1:** Baseline, demographic and manifestations of children with crescentic glomerulonephritis.

Variables	Total (*n* = 147)	Type I (*n* = 22)	Type II (*n* = 69)	Type III (*n* = 56)	*P* value
Age (years)	13.45 ± 3.75	13.21 ± 4.03	12.76 ± 3.41	14.19 ± 3.87	>0.05
Gender [males, *n* (%)]	65 (44.2)	13 (59.1)	36 (52.2)	16 (28.6)	>0.05
Hypertension	22 (15.0)	2 (9.1)	16 (23.2)	4 (7.1)	0.015^c^
Oliguria or anuria	120 (81.6)	20 (90.9)	48 (69.6)	52 (92.9)	0.045^a^, 0.001^c^
AKI, *n* (%)	131 (89.1)	22 (100.0)	60 (87.0)	49 (87.5)	>0.05
AKI 1 stage, *n* (%)	15 (11.5)	0 (0.0)	8 (11.6)	7 (12.5)	>0.05
AKI 2 stage, *n* (%)	31 (23.7)	2 (9.1)	19 (27.5)	10 (17.9)	>0.05
AKI 3 stage, *n* (%)	85 (64.9)	20 (90.9)	33 (47.8)	32 (57.1)	<0.001^a^, 0.004^b^
PCT, μg/L	0.68 (0.44, 0.84)	0.45 (0.18, 0.67)	0.70 (0.55, 0.81)	0.88 (0.54, 1.02)	0.043^a^, 0.008^b^
IL-6, ng/L	29.72 ± 6.24	22.07 ± 5.82	32.07 ± 6.24	41.44 ± 5.92	0.037^a^, 0.041^b^
Scr (mmol/L)	354.37 (78.52, 1,033.57)	536.44 (176.38, 1,033.57)	223.06 (76.19, 347.21)	338.56 (127.32, 426.06)	0.013^a^, 0.041^b^
eGFR, mL/min/1.73 m^2^	53.45 (16.00, 78.02)	25.07 (13.00, 48.02)	67.14 (24.43, 80.11)	47.38 (27.48, 67.04)	0.000^a^
Low C3	73 (49.7)	7 (31.8)	36 (52.2)	23 (41.1)	>0.05
Proteinuria (mg/kg•24 h)	36.52 ± 6.53	18.72 ± 4.04	43.36 ± 8.58	29.84 ± 5.54	<0.001^a^, 0.003^b^
Urine RBC, ul/ml	778.00 (275.00, 2,100.00)	1,100.00 (775.00, 2,300.00)	850.00 (277.00, 5,487.50)	424.50 (228.75, 1,025.25)	<0.001^b^, <0.001^c^
Urine NAG, U/g*cr	48.14 (31.10, 96.78)	86.10 (45.80, 106.93)	46.68 (36.24, 55.50)	38.60 (24.60, 72.10)	<0.001^a^, <0.001^b^
Urine RBP, mg/L	11.44 (3.00, 28.55)	29.00 (27.53, 31.32)	11.44 (3.26, 26.64)	7.80 (12.35, 26.32)	<0.001^a^, <0.001^b^
Anti-GBM positive, *n* (%)	24 (14.9)	22 (100.0)	0 (0.0)	2 (3.6)	–
ANCA positive, *n* (%)	61 (41.5)	2 (9.1)	5 (7.3)	54 (96.4)	<0.001^b^, <0.001^c^
MPO-ANCA positive, *n* (%)	58 (39.5)	2 (9.1)	5 (7.3)	51 (91.1)	<0.001^b^, <0.001^c^
PR3-ANCA positive, *n* (%)	5 (3.4)	0 (0.0)	0 (0.0)	5 (8.9)	–
Treatment					
Immunosuppressive therapy, *n* (%)	131 (89.1)	22 (100.0)	58 (84.1)	51 (91.1)	>0.05
CRRT, *n* (%)	91 (61.9)	19 (86.4)	27 (39.1)	45 (80.4)	<0.001^a^, <0.001^c^
ESKD three-month	13 (8.8)	5 (22.7)	3 (4.4)	4 (7.1)	0.008^a^
ESKD one-year	18 (12.2)	7 (31.8)	4 (5.8)	7 (12.5)	0.001^a^, 0.045^b^
ESKD five-year	53 (36.7)	11 (50.0)	17 (24.6)	26 (44.6)	0.025^a^, 0.011^c^
Dead five-year	7 (4.8)	3 (13.6)	2 (2.9)	2 (3.6)	>0.05

AKI, acute kidney injury; PCT, procalcitonin; IL-6, interleukin-6; Scr, serum creatinine; eGFR, estimate glomerular filtration rate; RBC, red blood cell count; NAG, N-acetyl-β-D-glucosidase; RBP, retinol-binding protein; Anti-GBM, anti-glomerular basement membrane; ANCA, antineutrophil cytoplasmicantibody-associated glomerulonephritis; CRRT, continuous renal replacement therapy; ESKD, end stage kidney disease.

Scr, AKI acute kidney injury.

^a^ *P* < 0.05 between types I and II.

^b^ *P* < 0.05 between types I and III.

^c^ *P* < 0.05 between types II and III.

### Pathological characteristics

Among the subtypes of CrGN, the scores for crescents, sclerosis, interstitial inflammation, and IF/TA in type II were lower than in type I and type III, respectively (*P* < 0.05, *P* < 0.01). The percentage of cellular crescentic in type I was higher than in type II and type III, respectively (*P* < 0.05, *P* < 0.01), and the fibrotic crescentic in type II was higher than in type I and type III, respectively (*P* < 0.05, *P* < 0.01). The tubulitis and arteriosclerosis scores in type III were higher than in type I and type II, respectively (*P* < 0.05, *P* < 0.01). Importantly, Bowman's Capsule Rupture (BCR) presented in 62 children (42.2%) with CrGN, and BCR in type I was higher than in type II and type III, respectively (*P* < 0.05, *P* < 0.01) (Table 2).

**Table 2:** Renal pathology data by type of crescentic glomerulonephritis.

Variables	Total (*n* = 147)	Type I (*n* = 22)	Type II (*n* = 69)	Type III (*n* = 56)	*P* value
Crescent score	1.81 ± 0.56	2.27 ± 0.49	0.96 ± 0.31	2.04 ± 0.52	0.008^a^, 0.012^c^
Cellular crescentic	25.00 (11.33, 49.17)	40.50 (34.58, 47.08)	21.37 (12.50, 56.47)	27.27 (12.78, 43.46)	0.007^a^, 0.044^b^
Fibrotic crescents	21.00 (9.85, 31.39)	28.50 (19.43, 43.81)	12.00 (7.00, 21.50)	22.50 (13.50, 33.75)	<0.001^a^, 0.022^c^
Sclerosis score	1.34 ± 0.56	1.37 ± 0.38	0.84 ± 0.37	1.54 ± 0.45	<0.001^a^, 0.004^c^
Bowman's capsule rupture, *n* (%)	62 (42.2)	15 (63.6)	23 (47.7)	24 (41.1)	0.004^a^, 0.044^b^
Interstitial inflammation score	1.66 ± 0.30	1.57 ± 0.32	0.94 ± 0.22	1.83 ± 0.25	0.036^a^, 0.041^c^
Tubulitis score	1.36 ± 0.52	1.22 ± 0.46	0.85 ± 0.32	1.56 ± 0.34	0.005^c^
TA/IF score	1.14 ± 0.22	1.18 ± 0.24	0.98 ± 0.18	1.36 ± 0.31	0.009^a^, 0.018^c^
Arteriosclerosis score	1.05 ± 0.36	0.98 ± 0.31	0.86 ± 0.24	1.25 ± 0.44	0.014^c^

IF/TA, intersititial fibrosis/tubular atrophy.

^a^ *P* < 0.05 between types I and II.

^b^ *P* < 0.05 between types I and III.

^c^ *P* < 0.05 between types II and III.

### Prognostic factors of renal outcome

**Figure 1:**
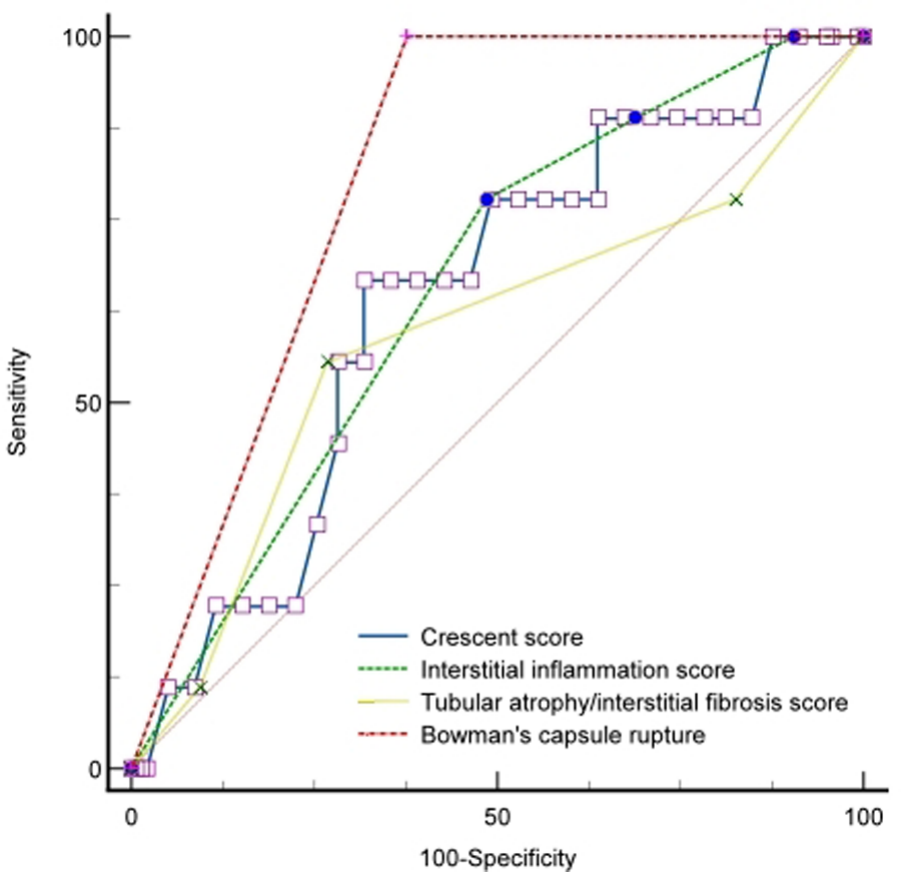
ROC curve of renal outcomes with the BCR, crescent, interstitial inflammation, and IF/TA scores.

We conducted Cox regression analyses to explore the clinical, laboratory, or histologic factors associated with ESKD. As shown in Table 3, multivariate analysis demonstrated that crescent score (HR = 2.185, 95% CI: 0.745–3.927, *P* = 0.033), interstitial inflammation score (HR = 5.164, 95% CI: 2.745–16.827, *P* = 0.037), IF/TA score (HR = 1.750, 95% CI: 1.018–3.009, *P* = 0.029), and BCR (HR = 8.874, 95% CI: 2.116–24.291, *P* = 0.012) were predictors of ESKD risk. The area under the curve (AUC) for ESKD prediction at the end of follow-up for the crescent score, interstitial inflammation score, IF/TA score, and BCR was 0.709 (*P* = 0.008), 0.657 (*P* = 0.031), 0.556 (*P* = 0.045), and 0.812 (*P* < 0.001), respectively. The best cutoff value for crescent score, interstitial inflammation score and IF/TA score were 2.45, 2.50 and 1.50, respectively, and the sensitivity and specificity of crescent score, interstitial inflammation score, IF/TA score and BCR were 66.7%, 77.8%, 55.6%, 100.0% and 71.7%, 51.5%, 73.2%, 62.3%, respectively. BCR showed better predictive value for ESKD than the interstitial inflammation score (*P* = 0.027) and IF/TA score (*P* = 0.047). However, there was no significant difference between the other factors (*P* > 0.05) (Figure 1).

**Table 3:** Risk factors of children progression to ESKD with CrGN.

Variables	Univariate analysis		Multivariate analysis	
	OR (95% CI)	*P*	OR (95% CI)	*P*
Scr (≥110 μmol/L)	10.237 (2.281–29.146)	0.048		
eGFR (≤15 ml/min/1.73 m^2^)	2.432 (1.326–14.758)	0.076		
CRRT	5.164 (1.033–8.673)	0.058		
PE	8.114 (0.152–17.146)	0.024		
Crescent score (per 0.01 increased)	3.225 (0.894–4.259)	0.011	2.185 (0.745–3.927)	0.033
Interstitial inflammation score (per 0.01 increased)	6.395 (3.064–18.191)	0.018	5.164 (2.745–16.827)	0.037
IF/TA score (per 0.01 increased)	2.386 (1.328–5.198)	0.007	1.750 (1.018–3.009)	0.029
Bowman's capsule rupture	12.352 (3.768–32.719)	0.002	8.874 (2.116–24.291)	0.012

Scr, serum creatinine; eGFR, estimate glomerular filtration rate; CRRT, continuous renal replacement therapy; PE, plasma exchange; IF/TA, intersititial fibrosis/tubular atrophy; BCR, Bowman's capsule rupture.

### Renal survival in short- and long-term

**Figure 2:**
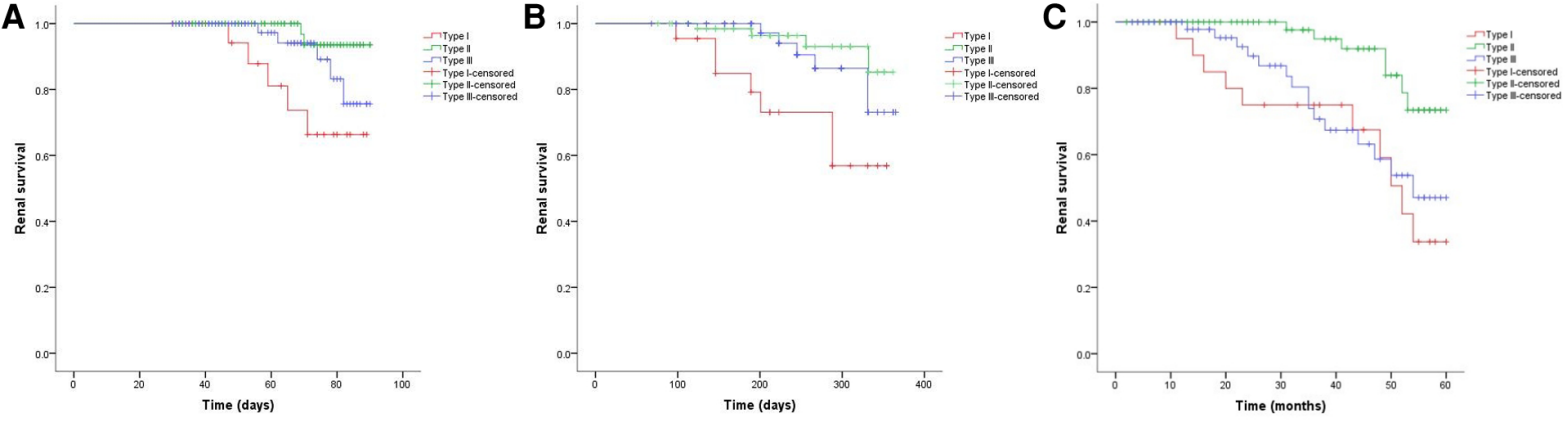
Cumulative renal survival of patients with different types of crescentic glomerulonephritis at 3-months (**A**), 1-year (**B**), and 5-year (**C**) follow-ups.

**Figure 3:**
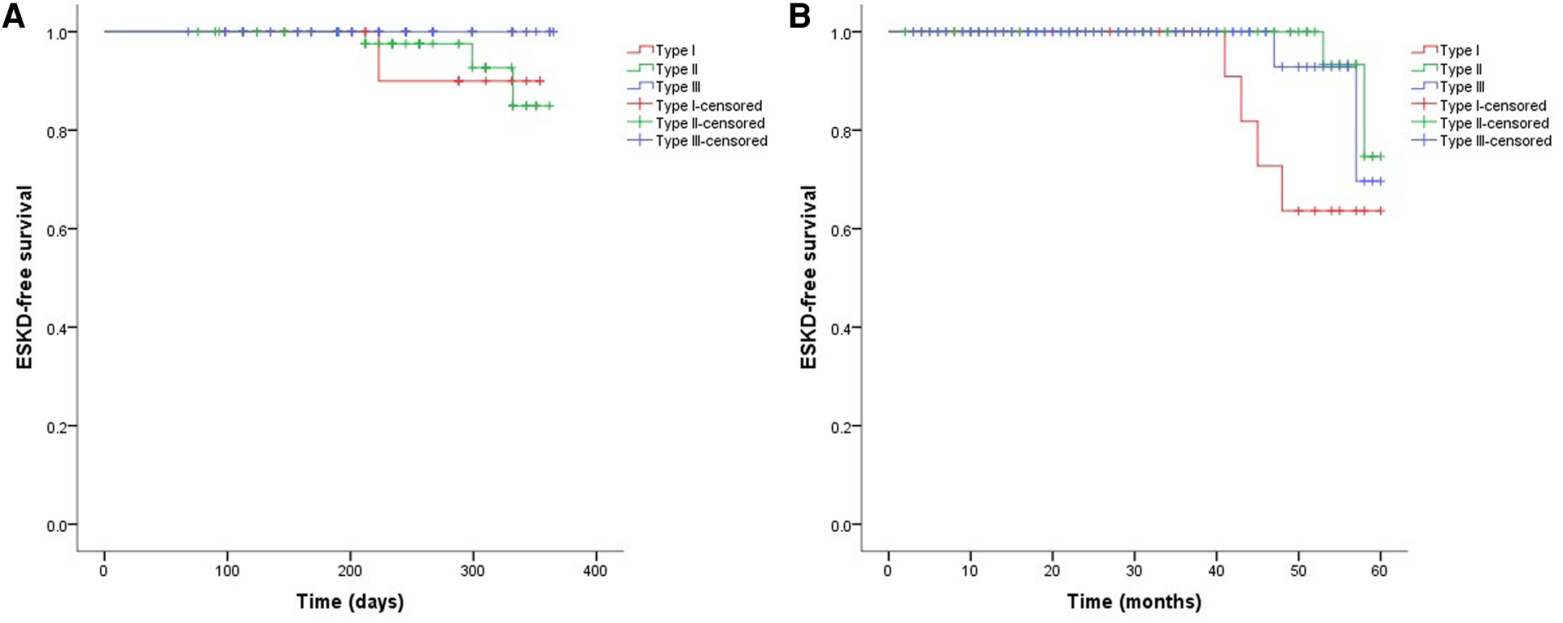
Cumulative ESKD-free survival in different types of crescentic glomerulonephritis at 1-year (**A**) and 5-year (**B**) follow-ups.

As shown in Figure 2, the 3-month cumulative renal survival rate for types I, II, and III CrGN was 66.3%, 93.6%, and 75.6%, respectively, and the rate in type I was significantly lower than in type II (*P* < 0.001). The 1-year cumulative renal survival rate for types I, II, and III CrGN was 56.9%, 85.3%, and 73.1%, respectively, and the rate in type I was significantly lower than in types II and II (*P* < 0.005, *P* < 0.001). The 5-year cumulative renal survival rate for types I, II, and III CrGN was 33.8%, 73.5%, and 47.1%, respectively, and compared with types I and III, type II of CrGN had a better renal recovery (*P* < 0.001) (Figure 3). The Kappa Consistency Test between the 3-month and 1-year total renal survival (82.1% vs. 74.7%) of the children was 0.683 (*P* < 0.001). After the 5-year follow-up, 7 children were dead, 3 children in types I and 3 children each in types II and III. The 5-year cumulative renal-free survival rate for types I, II, and III CrGN was 63.6%, 70.0%, and 69.6%, respectively, and the rate in type I was significantly lower than in type II (*P* < 0.005) (Figure 1). Within the 3-month follow-up, there were no deaths in the three types, and the Kappa Consistency Test between 1-year and 5-year total renal-free survival (78.3% vs. 69.1%) of the children was 0.476 (*P* < 0.001).

## Discussion

Validation studies of epidemiological data on CrGN had been performed in many countries, including Germany, Italy, Sweden, Turkey, Japan, and India, showing that CrGN accounted for 2.65% to 13% of total renal biopsies (9–13), and the date was 1.6%–3.7% in China (2, 14–16). There have been few reports of CrGN in children with large cohorts in the last 10 years (17–21). The 147 children with CrGN in our study accounted for 1.1% (147/1, 3,125, 1.1%) of renal biopsies in children during the same period, which was lower than in previous reports. Unlike adult studies in which type III CrGN was the most common histopathological form, immune complex glomerulonephritis was the most common cause of CrGN in children, commonly secondary to PIGN, HSPN, and IgAN. IgAN (15.6%) was the most common cause of type II CrGN, followed by LN (12.9%) and HSPN (9.5%) in our cohort. This small difference in epidemiological and etiology data may be related to the fact that our two institutes were tertiary care hospitals, and genetic and local factors may also affect the prevalence of the disease.

Of all the patients, type I CrGN showed more severe clinical manifestations than the other types, including AKI, PCT, IL-6, Scr, and eGFR. We also found different renal pathologic characteristics among the three subtypes. The scores for crescents, interstitial inflammation, and IF/TA in type II were lower than in type I and type III, respectively. Importantly, the percentage of BCR in type I was higher than in type II and type III. Multivariate analysis demonstrated that the crescent score, interstitial inflammation score, IF/TA score, and BCR were predictors of ESKD risk. A strong predictor of the outcome for all types of CrGN was the severity of kidney pathological lesions. Chen. et al. reported that pathological severity, represented by a higher crescent score, was associated with long-term outcomes (6). Zhao et al. found that acute tubular necrosis and Bowman's Capsule (BC) membrane thickening at presentation were independent risk factors for ESKD (22). In a South Asia survey, IF/TA and percentage of crescents were significant negative prognostic factors for patients with CrGN survival in the long term (23). Özlü. et al. demonstrated that the ratio of fibrous and/or fibrocellular crescents was inversely correlated with the response to treatment and development of ESKD (18), and fibrinoid necrosis and IF/TA were reported to be important risk factors for renal prognosis (24). Recently, histopathologic classification, chronic inflammation, and histopathologic vascular changes have been identified as a predictor for renal outcomes among CrGN patients (25, 26). Importantly, BCR showed better predictive value for ESKD than interstitial inflammation and the IF/TA score, and BCR was observed in 42.2% of children with CrGN, which demonstrates the importance of BCR in the pathological diagnosis and prognosis evaluation in CrGN and might be ignored in clinicopathology.

In the case of systemic and kidney-restricted diseases, the glomerular capillaries could develop lesions and necrosis, resulting in ruptures of the GBM and fibrin exudation. This process also triggers the activation of cellular and humoral components of inflammation in the BC, leading to the proliferation of parietal epithelial cells. The proliferation narrows the remaining space in the BC and presents as crescents on renal biopsy. BCR is the final step in crescent formation, and the proliferation of parietal epithelial cells during crescent formation leads to BCR (27). An intact BC prevents inflammatory cells from gaining access to the glomerular space. However, once the BC is breached, inflammatory cells could access the glomerular space in CrGN with BCR enabling direct pathological interaction between both compartments. When renal inflammation leads to the destruction of glomerular capillaries and the release of pro-inflammatory cytokines and chemokines into the BC, parietal epithelial cells proliferate and build new crescents (28). As the characteristic performance of CrGN, crescent formation has long been recognized as an indicator of the severity of the inflammatory process affecting the glomerulus. Our study also suggested that interstitial inflammation was a predictor of ESKD risk, which, accompanied by BCR, could be potential therapeutic targets.

In our cohort study, type I CrGN received more CRRT than type II and III, but the prognosis was not improved. Although prompt and intensive treatment measures were taken, including immunosuppressive therapy, CRRT, and plasma exchange (PE), 36.7% of the children progressed to ESKD at the 5-year follow-up. As CKD was defined as abnormalities of kidney structure or function, presented for >3 months (4), the endpoint of many AKI studies was 60- or 90-day mortality (29). ESKD at 1 year was a clinically important short-term outcome measure in patients with glomerulonephritis (GN) (30), and more than half of children with GN and crescents who progressed to ESKD did so within the first year after initial biopsy (17, 21). Therefore, we observed the short- and long-term renal and renal-free survival at 3-month, 1-year, and 5-year follow-ups, and the results showed that the Kappa Consistency Test between the 3-month and 1-year renal prognosis was 0.683 (*P* < 0.001), which indicated that the 3-month renal prognosis could partially reflect the 1-year renal prognosis. The result of the Kappa Consistency Test between 1-year and 5-year renal-free survival was 0.476 (*P* < 0.001), which indicated that the 1-year mortality rate could partially reflect the 5-year mortality rate of children with CrGN. From the association between 3-month and 1-year renal prognosis, we can infer that clinicians could predict early renal prognosis in children with CrGN, and aggressive therapy within 3 months was important for the recovery of renal function.

Limitations of this study: first, this study was a retrospective study of cases from two centers, and the trajectories identified in these participants may not be generalizable to other populations. Second, due to the limitations of retrospective studies, the choice of treatment may weaken the rigor of the study.

## Conclusions

In conclusion, AAGN, IgAN, and LN were the most common pathological types in children with CrGN in our cohort. The clinical manifestations, pathological change, and prognosis varied among different CrGN types. BCR, crescent, interstitial inflammation, and IF/TA scores were risk factors for the disease progressing to ESKD. The 3-month renal prognosis could partially reflect the 1-year renal prognosis, and the 1-year mortality rate could partially reflect the 5-year mortality rate of children with CrGN.

## Data Availability

The original contributions presented in the study are included in the article/Supplementary Material, further inquiries can be directed to the corresponding authors.
